# Singling out the double effect - sexual health advice and contraception are ethically distinct

**DOI:** 10.1080/17571472.2015.1082341

**Published:** 2015-09-28

**Authors:** Steven Bow

**Affiliations:** ^a^ Public Health Department, London Borough of Richmond upon Thames, Twickenham, UK

**Keywords:** Sexual health advice, contraception, contraceptives, condoms, ethics, doctrine of double effect, health care workers, primary care, general practice, Thomas Aquinas, pregnancy, good, evil, Gillick, Fraser guidelines

## Abstract

This article is a response to an article previously published in LJPC, which employed the doctrine of double effect to explain the Gillick judgement and exculpate health care workers who provide contraceptives and sexual health advice to under-16s. In this analysis, the two acts: provision of contraceptives and provision of sexual health advice are examined separately against the four criteria of the doctrine of double effect. In conclusion, whilst sexual health advice provision fits into the doctrine reasonably well, in the case of contraceptive provision, the validity of the doctrine of double effect is more doubtful.

## Key messages

• Whilst sexual health advice provision fits into the doctrine of double effect reasonably well, in the case of contraceptive provision, the validity of the doctrine of double effect is more doubtful.
• Some health care workers will not agree that the provision of contraception is a morally neutral act.
• The benefits of sexually transmitted disease and pregnancy prevention cannot be realised unless underage sex actually occurs.
• Prevention of pregnancy is implicitly counted as a benefit. However, in and of itself, pregnancy is not a harm.

## Why this matters to me

As a public health professional, sexual health interventions fall within my sphere of interest. However, there is a natural tendency to focus on the health and mental well-being of the populations receiving an intervention and it is easy for the strategy to be developed at a distance from front-line staff. Nevertheless, it is important that equal, or even greater importance, is given to the mental and moral well-being of those staff who are expected to carry out public health interventions in primary care.

## Introduction

In the UK, the Sexual Offences Act 2003 criminalises sexual activity involving anybody under the age of 16.[1] By extension, ‘aiding, abetting, counselling or procuring the commission’ of underage sex is also an offence. However, youth workers, doctors, nurses and other health care workers (henceforth simply referred to as health care workers, or HCWs for convenience) are frequently called on to provide contraceptives or sexual health advice to under-16s, with the intention of encouraging ‘safe sex’.[2] Such provision clearly falls within the ambit of aiding, abetting and counselling the commission of underage sex. In removing the major tangible disincentives of sex, the HCW is assisting the act itself. In recognition of these implications, the Sexual Health Act makes an exception (Section 73) for those acting to protect a child’s safety, prevent sexually transmitted infection or pregnancy, or promote their emotional well-being by the provision of advice.

This pragmatic approach may *legally* exculpate providers of sexual health advice, but are they excused *ethically*? This question mark could hang heavily over the conscience of a HCW. In an article in LJPC, Papanikitas sought to answer this question, using the long-established *doctrine of double effect.*[3] The article focused on general practice, reflecting the ambition for doctors’ decisions to be not merely lawful, but ethical too.[4] However, the argument Papanikitas employs is equally applicable to other professionals and non-professionals.

The *doctrine of double effect* (hereafter, the doctrine) is a line of ethical reasoning that can be traced back to St Thomas Aquinas and relies on distinguishing between the intended and foreseeable but not intended consequences of an action (hence ‘double’ effect).[5] How do we distinguish what is foreseeable from what is intended? Many actions have multiple foreseeable consequences, but, so the argument goes, we are only morally tied to the particular consequence which we intended; the driving purpose of the action. It is typically formulated along the following lines, with four criteria which must be fulfilled for the principle to hold true.

The ‘Doctrine of Double Effect’ states that a person may in good conscience perform an action that he foresees will produce a good and a bad effect, provided that:

(1) The action itself is not evil (i.e. is good or, at least, indifferent).
(2) Only the good effect is intended.
(3) The good effect does not proceed from the evil effect.
(4) There is a proportionately grave reason for permitting the evil effect.

Papanikitas proposes that the doctrine can be deployed in this instance, on the grounds that there are two effects of the HCW’s intervention; one intended (protecting a minor from the harms of unwanted pregnancy and STIs) and one foreseen but not intended (assisting underage sex). He argues that the doctrine’s four criteria apply in this type of situation, and more readily so than in other areas in which it is frequently invoked (such as the provision of medication intended for pain relief at the end of life that may incidentally hasten death). He concludes that the doctrine can indeed be used to ethically justify the provision of contraceptives and sexual health advice to minors.

The discourse takes place in the context of the widely cited guidelines articulated by Lord Fraser in the case of Gillick vs West Norfolk^1^ and Wisbech Area Health Authority and the Department of Health and Social Security’s ensuing national guidance.[7] Papanikitas argues that the Fraser guidelines implicitly reflect the distinction between intention and foresight.^2^

## Scrutiny of double effect conditions

In writing this response, I do not discard the arguments that Papanikitas proposed, but examine their objects more closely. Although bundled together in the Fraser guidelines as ‘contraceptive advice and treatment’ and in the subsequent Department of Health guidelines as ‘advice or treatment … on contraception, sexual and reproductive health’, contraception and sexual health advice are distinct, both practically and ethically. Practically, sexual health advice is constituted of words, whilst contraception is embodied in a medication or appliance. In what follows I intend to show how they also diverge ethically when each of the doctrine of double effect criteria is applied.

The first criterion of the doctrine is the moral valence of the action in question: it must be neutral or good. In other words, not evil. Provision of sexual health advice of itself is morally neutral as long as the information conveyed is tolerably complete, unbiased and comprehended. It is likely that, in reality, sexual health advice falls short of these criteria by varying degrees. Notwithstanding this, for the sake of brevity I use the phrase ‘sexual health advice’ as shorthand for ‘complete, unbiased and comprehended medical information about sexual intercourse, contraceptive methods and reproduction’.

It is taken as read that the advice discourages underage sex. However, whilst actively encouraging underage sex may commonly be deemed criminal practice, the provision of information without actively discouraging underage sex could be considered morally neutral. Nonetheless, the argument does not rest upon this proposition.

Provision of contraceptives, on the other hand, is more controversial than simply passing on knowledge. Traditional morality held that contraception was illicit and, despite widespread softening of attitudes during the twentieth century, a degree of opposition remains.[8,9] It is beyond the scope of this article to debate the ethical reasoning for or against contraception. However, it is likely that, in the UK, a significant minority of healthcare workers have qualms about the morality of contraception in itself, and, for them, this criterion is not fulfilled.[10–12] Therefore, for them, the doctrine of double effect does not provide moral justification for providing contraceptives.

Papanikitas accedes this point as a brief caveat to his paper and, in his brevity, equates condemnation of contraception with the exaltation of pregnancy. Although the former is rarely found without the latter, we must not confuse actions with consequences. This criterion of the doctrine of double effect is only concerned with the act and it is the next criterion that is concerned with the consequences.

The second criterion of the doctrine is that, of the multiple foreseeable effects of the action, only the good effect is intended by the actor. The generally intended effect of both sexual health advice and contraception is twofold: to prevent STIs and pregnancy. For the doctrine of double effect to hold true, both these effects must be demonstrably good (or at least neutral).

STIs are, unequivocally, a harm in themselves, so STI prevention must be a good effect. This conforms to the doctrine. It is, of course, relevant to note that STIs may not be prevented by either provision. Whilst sexual health advice, as information, may indeed lead a teenager to take fewer risks and thus be protective against STIs, many forms of contraception do little to protect against disease, and none are proof against all STIs.[13]

The arguments employed in the Gillick judgement, however, dwell on unintended pregnancy as the principal harm, which is articulated in three forms. The first is the fact of childbirth to an ‘immature and irresponsible mother’, making such a pregnancy ‘wholly undesirable’ (p.33, Lord Bridge of Harwich). Although this sentiment may be easy to accept, on scrutiny it is not immediately clear who is thought to be afflicted with this harm – the unintended child, the irresponsible mother or wider society – nor what the precise nature of the harm might be to each.

The 1980 Department of Health and Social Services’ (DHSS) guidance cited in the judgement also mentions ‘long-term physical, psychological and emotional consequences which are equally a threat to stable family life’. It is not clear if these are thought to be caused by STIs, pregnancy or both. In the case of pregnancy, these adverse consequences might plausibly be related to the financial, emotional and social burden on the mother of raising a child. However, whilst they may be felt more acutely, none of these are unique to under-16s. If these consequences dictate that pregnancy be considered a harm for under-16s, then they logically render every pregnancy a harm. Unintended pregnancy is also linked, by Scarman and Bridge, to abortion, as a likely or presumed outcome. Therefore, the emotional impact of abortion is seen as a referred harm of unintended pregnancy. This consequence is rather distanced from the initial act and would be mediated by many other factors. For example, Mrs. Gillick also sought assurances that abortion treatment would not be given without parental knowledge either. The tenuousness of abortion as a direct harm is harder to interpret ethically.

Defining prevention of pregnancy as a good effect is, therefore, problematic. Pregnancy is not usually considered to be a harm in itself. Prefixing something with the adjective ‘unwanted’ or ‘unintended’ is insufficient to make it evil. As mentioned above, Papanikitas connects this argument with a particular moral stance against contraception. However, in an ethical, medical or philosophical sense, can conception, gestation and birth be considered true evil effects?

The negative outcomes listed above may well be considered ‘evil’. But, for some, they are not corollaries, that is, not necessary consequences, of pregnancy. For the others, the suggested intervention does not negate them. Underage sex that does not result in a child will not make an irresponsible girl a responsible girl (although having a child just might make an irresponsible girl a responsible mother).

The third criterion is that the good effect must be independent of the evil effect; it must not proceed from it.

The benefits of sexual health advice are truly independent of whether underage sex occurs. If it does occur, it will at least be well informed. And if not, as Papanikitas states, ‘the benefit of the advice is not lost’; knowledge remains.

However, for contraception, when we thumb along the links of the chain of causation, it is a different story. For, simply put, contraception is useless without sex. Unless underage sex occurs, a contraceptive pill, implant or barrier is impotent. Contraception cannot prevent conception (the intended effect), without coitus being antecedent (the purported evil effect). This violates the third criterion of the doctrine as the good effect proceeds from the evil effect.

The final criterion of the doctrine of double effect requires the good effect to be sufficiently good to justify the evil effect. This ultimately resolves to be a subjective judgement, which requires weighing the values of the consequences on our own personal set of scales. This is true when applying the doctrine to any ethical situation; however, it is particularly knotty in the case of contraception. On one arm of the scale might be placed STI and pregnancy prevention, and on the other arm are placed the evil effects.

The principle evil effect in question goes back to the issue identified by the Sexual Health Act: that contraception and sexual health advice provide tacit assistance and even acceptance of underage sex. As Lord Brandon argues, in the case of a man and an under-16 girl,

To give the girl contraceptive treatment, following appropriate advice and examination, is to remove largely [the inhibition of the risk of unwanted pregnancy]. Such removal must involve promoting, encouraging or facilitating the having of sexual intercourse between the girl and the man.

Even in this formulation, the ethical decision hinges on the HCW’s relative evaluation of STI prevention, pregnancy prevention, underage sex and what constitutes ‘proportional gravity’. It is not easy to anticipate the outcome of such a calculation.

As already discussed, the moral valence of pregnancy prevention is debatable, and HCWs may differ on the matter. If prevention of pregnancy is not seen as a good effect, and is removed from the scales, or even switches sides, it is clear the impact that this may have on the final ethical judgement.

## Conclusion

Therefore, separating out the two acts which have been hitherto conflated, we can see their ethical differences. In accordance with Papanikitas’ assertion, a HCW providing sexual health advice to an under-16 can justify their action in terms of the doctrine of double effect. Their advice is ethically neutral and they only intend its good effects. The good effects do not rely on underage sex taking place and can be reasonably judged to be proportionate.

Nevertheless, the morality of their action also hinges on their evaluation of pregnancy (and, thus, prevention thereof) and depends on the degree to which their advice is complete, unbiased and comprehended.

On the other hand, upon scrutiny, the provision of contraceptives cannot be formulated according to the doctrine of double effect. At a minimum, it fails the third criterion of the doctrine, as any benefit of contraception cannot be realised without underage sex taking place. Furthermore, the ethical ambivalence of the act of contraception and the outcome that is pregnancy, mean that the other three criteria are also in doubt. Therefore, a HCW providing an under-16 with contraception in accordance with the Sexual Health Act must look for ethical justification from someone other than St Thomas; perhaps in the direction of consequentialist philosophers Bentham and Mill.

## Ethical approval

N/A

## Conflicts of interest

None

## Disclosure statement

No potential conflict of interest was reported by the author.

## Footnotes

^1^ Gillick v West Norfolk and Wisbech AHA [1986] AC 112, [1985] 3 All ER 402 (HL): Mrs. Gillick sought a declaration that, whilst under 16, her daughters would not be provided any contraceptive or abortion advice or treatment without parental/guardian consent. It was escalated to the House of Lords, which ruled that minors could consent to such treatment if they understood the treatment offered.

^2^ The Fraser guidelines for provision of contraception or sexual health advice without parental consent: ‘the young person understands the health professional’s advice; the health professional cannot persuade the young person to inform his or her parents or allow the doctor to inform the parents that he or she is seeking contraceptive advice; the young person is very likely to begin or continue having intercourse with or without contraceptive treatment; unless he or she receives contraceptive advice or treatment, the young person’s physical or mental health or both are likely to suffer; the young person’s best interests require the health professional to give contraceptive advice, treatment or both without parental consent’ (Op. cit. Department of Health, 2004).
